# A Preclinical Validation of Iron Oxide Nanoparticles for Treatment of Perianal Fistulizing Crohn’s Disease

**DOI:** 10.3390/ijms23158324

**Published:** 2022-07-28

**Authors:** Antoine Cazelles, Maxime K. Collard, Yoann Lalatonne, Sabrina Doblas, Magaly Zappa, Camélia Labiad, Dominique Cazals-Hatem, Léon Maggiori, Xavier Treton, Yves Panis, Ulrich Jarry, Thomas Desvallées, Pierre-Antoine Eliat, Raphaël Pineau, Laurence Motte, Didier Letourneur, Teresa Simon-Yarza, Eric Ogier-Denis

**Affiliations:** 1 Département of Chirurgie Colorectale, Assistance Publique Hôpitaux de Paris, Hôpital Beaujon, CEDEX, 92110 Clichy, France; antoinecazelles@hotmail.fr (A.C.); maximecollard1990@gmail.com (M.K.C.); camelia.labiad@gmail.com (C.L.); leon.maggiori@aphp.fr (L.M.); yves.panis@aphp.fr (Y.P.); 2 Centre de Recherche sur l’Inflammation, INSERM, U1149, CNRS, ERL8252, Université Paris Cité, Team Gut Inflammation, BP 416, 75018 Paris, France; dominique.cazals-hatem@aphp.fr (D.C.-H.); xtreton@gmail.com (X.T.); 3 Laboratory for Vascular Translational Science, Université Paris Cité, Université Sorbonne Paris Nord, LVTS, INSERM, UMR 1148, 75018 Paris, France; yoann.lalatonne@aphp.fr (Y.L.); laurence.motte@univ-paris13.fr (L.M.); didier.letourneur@inserm.fr (D.L.); teresa.simon-yarza@inserm.fr (T.S.-Y.); 4 Départements of Biochimie and de Médecine Nucléaire, Assistance Publique-Hôpitaux de Paris, Hôpital Avicenne, 93009 Bobigny, France; 5 Centre de Recherche sur l’Inflammation, INSERM, U1149, CNRS, ERL8252, Laboratory of Imaging Biomarkers, Université Paris Cité, BP 416, 75018 Paris, France; sabrina.doblas@inserm.fr (S.D.); magaly.zappa@aphp.fr (M.Z.); 6 Département of Radiologie, Assistance Publique Hôpitaux de Paris, Hôpital Beaujon, CEDEX, 92110 Clichy, France; 7 Département of Pathologie, Assistance Publique Hôpitaux de Paris, Hôpital Beaujon, CEDEX, 92110 Clichy, France; 8 Département Gastroentérologie, Assistance Publique Hôpitaux de Paris, Hôpital Beaujon, CEDEX, 92110 Clichy, France; 9 Université Rennes, CNRS, INSERM, BIOSIT UAR 3480, US_S 018, Oncotrial, 35000 Rennes, France; ulrich.jarry@univ-rennes1.fr (U.J.); thomas.desvallees@univ-rennes1.fr (T.D.); 10 Biotrial Pharmacology, Unité De Pharmacologie Préclinique, 35000 Rennes, France; 11 Université Rennes, CNRS, INSERM, BIOSIT UAR 3480, US_S 018, PRISM, 35000 Rennes, France; pierre-antoine.eliat@univ-rennes1.fr; 12 INRAE, INSERM, Institute NUMECAN, UMR_A 1341, Université Rennes, UMR_S 1241, 35000 Rennes, France; 13 INSERM, CLCC Eugène Marquis, Oncogenesis, Stress Signaling, Université Rennes, UMR_S 1242, 35000 Rennes, France; raphael.pineau@inserm.fr; 14 INSERM U1242, Centre Eugène Marquis, Rue de la Bataille de Flandres-Dunkerque, 35042 Rennes, France

**Keywords:** Crohn’s disease, perianal fistula, preclinical model, iron oxide nanoparticles, fistula treatment

## Abstract

Fistulizing anoperineal lesions are severe complications of Crohn’s disease (CD) that affect quality of life with a long-term risk of anal sphincter destruction, incontinence, permanent stoma, and anal cancer. Despite several surgical procedures, they relapse in about two-thirds of patients, mandating innovative treatments. Ultrasmall particles of iron oxide (USPIO) have been described to achieve in vivo rapid healing of deep wounds in the skin and liver of rats thanks to their nanobridging capability that could be adapted to fistula treatment. Our main purpose was to highlight preclinical data with USPIO for the treatment of perianal fistulizing CD. Twenty male Sprague Dawley rats with severe 2,4,6-trinitrobenzenesulfonic acid solution (TNBS)-induced proctitis were operated to generate two perianal fistulas per rat. At day 35, two inflammatory fistulas were obtained per rat and perineal magnetic resonance imaging (MRI) was performed. After a baseline MRI, a fistula tract was randomly drawn and topically treated either with saline or with USPIO for 1 min (*n* = 17 for each). The rats underwent a perineal MRI on postoperative days (POD) 1, 4, and 7 and were sacrificed for pathological examination. The primary outcome was the filling or closure of the fistula tract, including the external or internal openings. USPIO treatment allowed the closure and/or filling of all the treated fistulas from its application until POD 7 in comparison with the control fistulas (23%). The treatment with USPIO was safe, permanently closed the fistula along its entire length, including internal and external orifices, and paved new avenues for the treatment of perianal fistulizing Crohn’s disease.

## 1. Introduction

Crohn’s disease (CD) belongs to the group of inflammatory bowel diseases (IBD), of which the main inflammatory sites are the small intestine, colon, anus, and perineum. Anoperineal lesions (APLs) are frequent and particularly difficult to treat because of the induced tissue destruction and their recurrence. APLs are classified as non-fistulizing lesions (skin tags, fissures, ulcers, anorectal stricture, and anal cancer) and fistulizing lesions (simple or complex perineal fistulas, rectovaginal fistulas ± abscessed) [1]. Fistulizing anoperineal lesions (FAPLs) are the most severe ones, with a long-term risk of anal sphincter destruction, incontinence, permanent stoma, and anal cancer. The management of FAPLs is complex and multimodal, requiring the complementary expertise of gastroenterologists, radiologists, nutritionists, and surgeons. More than two-thirds of patients have an abscess associated with FAPLs; thus, the treatment first includes a surgical phase of examination, abscess drainage, and insertion of a seton through the fistula tract. The second phase is medical treatment, using TNF alpha antagonists in order to control disease-related inflammation. A final surgical phase aiming to close the fistula tract may be considered in a symptomatic patient with no concomitant abscess and with medically controlled proctitis [2]. Several surgical procedures are available to close the fistula tract: fibrin glue, chronic seton, endorectal advancement flaps, plug, ligation of the intersphincteric fistula tract, and video-assisted anal fistula treatment; however, their efficacy is limited (no more than 30% of healing at 1 year) [2,3]. FAPLs relapse in about two-thirds of patients, leading to a repetition of surgical procedures, including for patients with uncontrolled disease, diverting stoma, or proctectomy. Recently, the local injection of mesenchymal stem cells has been investigated with regard to fistula closure [4,5,6,7] and is now proposed as a therapeutic option in the latest ECCO (European Crohn’s and Colitis Organisation) recommendations [8]. However, there are many limitations in stem cell-based therapy: the tissue origin of stem cells (there are no studies comparing side by side the use of adipose- vs. bone-marrow-derived mesenchymal stem cells), autologous or allogenic stem cells, number of cells, logistic and regulatory hurdles for the production site and hospital, short shelf life of cells, low availability of treatments, and expensive costs. Therefore, innovative and more affordable treatments are mandated to maintain the long-term closure of FALPs and the remission of perianal fistulizing CD.

Recently, we developed the first reliable and reproducible preclinical model of a trans-sphincteric perianal fistula with pathological inflammation of the rectum that closely reproduces the pathological characteristics of the perianal fistula in CD [9]. Interestingly, a promising regenerative medicine using ultrasmall particles of iron oxide (USPIO) has also been proposed by some of us [10,11]. Nanobridging (adhesion by aqueous nanoparticle solutions) was applied in vivo to achieve with USPIO the rapid and strong closure and healing of deep wounds in the skin and liver in rats. Another advantage is that nanobridging brings the edges of the tissue together, favoring the physiological reparative process, whereas synthetic polymers, when polymerizing, usually form a rigid interface that does not allow contact of the edges; thus, this interferes with the repair process. Furthermore, iron oxide nanoparticles have already been used intravenously as a contrast agent for quantifying arterial wall inflammation by MRI in preclinical and clinical settings [12,13,14]. In vivo studies have shown that after penetrating the cells, iron oxide nanoparticles remain in cell organelles (endosomes/lysosomes), contribute to cellular iron pool, and release into the cytoplasm after decomposing [15].

The main purpose of this research was to highlight preclinical data for the treatment of perianal fistulizing CD with USPIO.

## 2. Results

**Figure 1:**
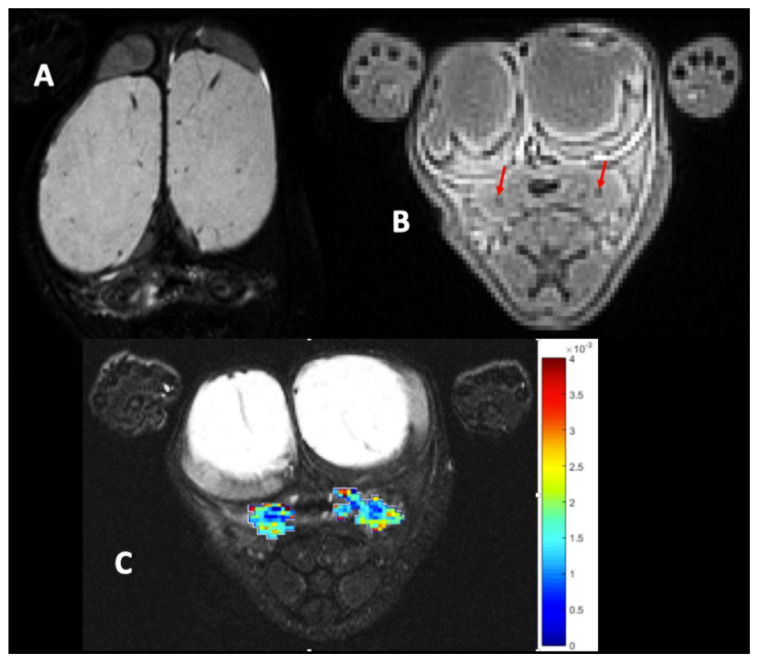
Baseline MRI. (**A**): T2-weighted image, axial section, hyposignal of the fistula tract, and hypersignal of peripheral inflammation. (**B**): Axial ultra-short echo time (UTE) images evidenced the 2 visible tracks (red arrows). (**C**): Inflammation is seen as a high apparent diffusion coefficient (ADC) area on the ADC map. The ADC scale is located on the right.

The preclinical study on 20 rats was 42 days long, and was performed on the basis that two fistulas were made per individual. All the rats had clinical signs of colitis, including bleeding and soft stools and perineum tumefaction on day 7 after a 2,4,6-trinitrobenzenesulfonic acid solution (TNBS) enema. While threads were maintained for 28 days, all the rats were anaesthetized 8 times on average (min 6–max 11), in order to ensure the threads were present, and twice a week to instill 100 µL of TNBS solution in each fistula tract. New threads were reinserted 6 times on average during this period (min 0–max 11). Twelve (70%) rats developed an abscess during this phase, which healed in about 1 week. Still during this phase, three rats tore out one of the two threads, causing a fistulotomy. Those three individuals with only one fistula tract were excluded from the global comparative study. Two of them were treated with USPIO and one rat was treated with a saline solution. They were kept alive until post-operative day (POD) 14 in order to assess the long-term fate of the USPIO suspension. The fistulas’ characteristics before treatment of the 17 rats composing the main analysis are summarized in Table 1. On clinical and radiological assessments, all 17 rats presented two inflammatory fistulas before treatment. Fifteen rats had a chronic proctitis graded at 1, and two rats had no detectable proctitis before treatment. The mean MRI-calculated diameter of the fistula tracts was 2.17 mm (±0.6 [1.2–3.3]) for the treated fistulas and 1.92 mm (±0.3 [1.5–2.5]) for the control fistulas. The baseline MRI images are shown in Figure 1. Similar results were observed for the three excluded rats, as detailed in Table 2.

### 2.1. Control Fistulas

As presented in Table 3 and Table 4, the control fistulas had a low and poor natural healing process over time. At POD 7, only 4 (23%) tracts had an internal orifice closed on the MRI, and 12 (71%) were still visualized on more than 50% of their tract. Only one control fistula tract was mainly closed at POD 7. All the control fistulas had peripheral tract inflammation until POD 7. These results are similar to those previously published for the development of the model of perianal fistulizing Crohn’s disease [9].

### 2.2. Fistulas Treated with the Ultrasmall Particles of Iron Oxide

**Figure 2:**
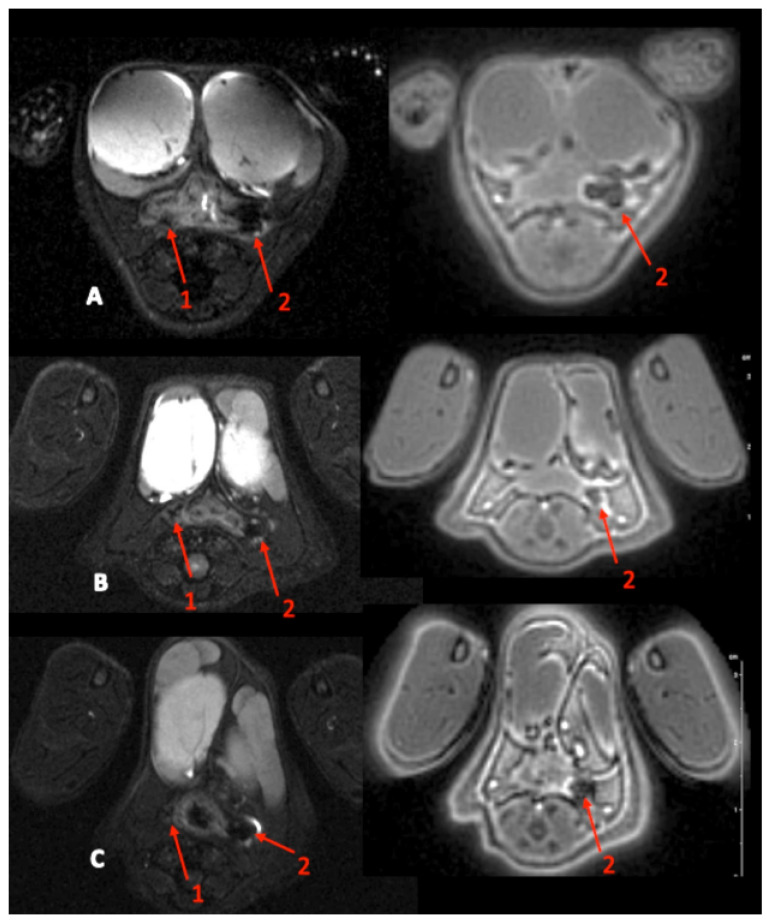
MRI images at POD 1 (**A**), POD 4 (**B**), and POD 7 (**C**). Left: T2-weighted image axial section. Right: axial ultra-short echo time (UTE) image. Red arrows 1: control fistula. Red arrows 2: fistula treated with USPIO (on the T2-weighted images, note the presence of an MRI hyposignal due to the iron nanoparticles present in the tract).

As presented in Table 3, clinical examination of the treated fistulas showed that all external and internal orifices were closed by USPIO at POD 7. The MRI findings are shown in Table 4 and the images in Figure 2. The MRI assessment showed that all the treated fistula tracts were permanently filled or closed with USPIO up to POD 7. All the external and internal orifices were closed until POD 7. The treated tracts diameters were overestimated by the MRI due to the blooming effect caused by the presence of iron oxide nanoparticles. The observed diameters seemed to decrease over time, highlighting an active metabolism of nanoparticles. Peripheral tract inflammation could not be measured because of the nanoparticles’ MRI artefact. The same results with USPIO were observed until POD 14 (Table 2).

### 2.3. Pathological Examination

**Figure 3:**
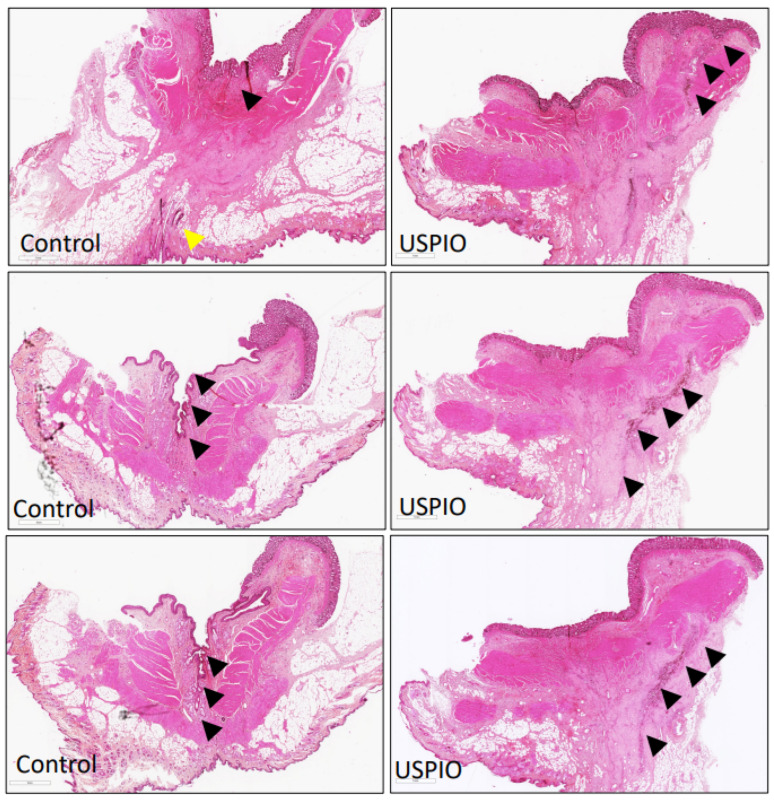
Histological characteristics of the control and USPIO-treated fistulas: representatives H&E-stained serial slices showing control (**left** panels) and USPIO-treated (**right** panels) specimens (magnification ×100). The control fistula lumen was visible with internal (black arrowhead—digestive side) and external (yellow arrowhead—perineal skin) orifices. There were local inflammatory signs of suppurative inflammation with abscess formation around the epithelialized fistula. The USPIO-treated transphincteric fistula was closed on its entire length (black arrowheads—revealed by black staining in the H&E section). Note that the internal orifice was no longer visible.

Serial histological sections of the control fistulas taken at POD 7 showed an open, transphincteric, and epithelialized fistula tract. The presence of moderate to severe acute and chronic inflammation was detected at the periphery of the saline-treated fistula by neutrophil and granulation tissue, and lymphoplasmocyte infiltration, respectively. USPIO treatment closed the entire length of the tract and facilitated the closure of the internal orifice with fibrous scarring (Figure 3).

### 2.4. Safety

Throughout our preclinical study, no rat died and all the rats maintained a good general condition without any weight loss. The USPIO administration was very simple and did not require any invasive procedure. One rat treated with USPIO developed an abscess at POD 1 that spontaneously healed in 1 week. No other adverse effect was reported. Because the liver is usually the dominant organ for the clearance of the iron oxide nanoparticles [16,17], at POD 7, we evaluated liver function (transaminases, alkaline phosphatases, and bilirubin) and iron concentration assessments. All the biological results were in the normal range (data not shown). The same results were observed at POD 14 with the two rats treated with USPIO on the single tract.

## 3. Discussion

Our preclinical study showed that the USPIO treatment allowed the closure and/or filling of all the treated fistulas from its application until POD 7. These results were assessed by clinical examination and by MRI. In contrast, no control fistula was closed on POD 1. Despite natural healing, only 1 control fistula was completely closed at POD 7, and 12 (71%) were still open for more than 50% of their length. The potential anti-inflammatory effect of early and effective tract closure could not be assessed on the MRI due to the “blooming” effect of iron oxide making peripheral inflammation non-measurable. In our hands, treatment with USPIO was simple and safe: one rat developed an abscess the day after treatment, which spontaneously recovered within one week. No other adverse effects were observed. Despite a mainly hepatic metabolism, iron oxide nanoparticles had no effect on liver function (transaminases, alkaline phosphatases, and bilirubin) and physiological iron concentrations.

This study was the first one to evaluate the use of our preclinical model for therapeutic investigations. The control fistulas suggested that our model is reliable and reproducible. It is perfectly suitable in practice for testing new therapies. The internal and external orifices and fistula tracts are well-calibrated. The MRI results indicated that all the control tracts have persistent peripheral inflammation until POD 7. These results confirm those of the initial published models [9]. Some other experimental models of FAPLs exist in different species, including rats, pigs, and rabbits [18,19,20,21,22]. However, none of these models mimic fistulizing perianal Crohn’s disease since they do not exhibit proctitis or peripheral inflammation of the fistula tract. In CD, a strong association between colorectal inflammation and FAPLs is clearly established [23] and the presence of a fistula constitutes an aggravating factor of the disease. Therefore, preclinical studies on Crohn’s FAPLs cannot be considered without an inflammatory context.

However, our study has some limits. The first limitation is the limited long-term data on the evolution of the USPIO within the fistula tracts. We evidenced here the efficiency of USPIO treatment on fistula closure until POD 7 by MRI and histopathology, demonstrating that USPIO were still present at this time. Furthermore, from the three rats we excluded, comparable MRI results to those obtained at POD 14 showed durable closure of the USPIO-treated fistula tract. A second limitation is that our experimental model only concerns simple transphincteric fistulas and does not concern complex fistulas; this does not allow us to conclude on the efficacy of this treatment on these complex tracts. However, the results obtained and the mode of administration of this treatment give hope of the effectiveness of USPIO on FAPLs.

This study suggests that topical USPIO treatment might be a safe and efficient treatment of FAPLs. Regarding the feasibility of a clinical study, USPIO seem perfectly adapted for fistula treatment. They can be easily prepared, preserved at an ambient temperature for long time, and have been already produced in GMP forms for clinical studies. They can be easily applied by a flexible syringe and we only need to maintain sufficient pressure for 1 min. Consequently, USPIO seem to be a promising new treatment of perianal fistulizing Crohn’s disease.

## 4. Materials and Methods

### 4.1. Study Design and Animals

**Figure 4:**
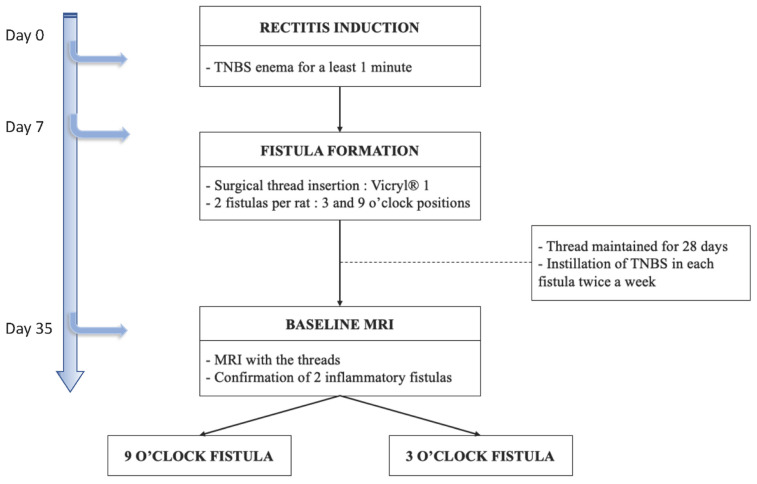
Scheme of the timeline and the main steps for inflammatory fistulas formation from day 0 to day 35.

A preclinical study testing USPIO for the treatment of a perianal fistula was performed in a rat model of perianal fistulizing Crohn’s disease developed in our laboratory [9]. This experimental model develops a perianal fistula with pathological inflammation of the rectum, characterized by a persistent fistula tract after more than 7 days. Two fistulas by rat were generated, allowing that each rat was its own control. The main steps of inflammatory fistula formation are summarized in Figure 4. The animals were male Sprague Dawley rats aged between 42 and 90 days. They were bred and housed in a conventional area, fed with irradiated food and hydrated orally ad libitum, including the day before and the day of the procedure. Up to two rats were housed in the same cage. The rats were weighed on the day of each procedure and then every 7 days. All the experiments were performed in compliance with the European Community guidelines and approved by the Institutional Animal Care and Use Committee (no. APAFIS#23031-2019112613522589v5 Paris Nord ethics committee and French Research Ministry).

All the procedures were performed according to the same anesthetic and analgesic protocol. The animals were anaesthetized with isoflurane inhalation (isoflurane 3 L/min + O_2_ 2 L/min during induction, then isoflurane 1.5 L/min + O_2_ 2 L/min). Analgesia was performed by local injection of 20% lidocaine hydrochloride (1 mg/kg) and by subcutaneous abdominal injection of buprenorphine (0.05 mg/kg/rat). Post-operatively, if the animal showed any signs of pain-related stress, buprenorphine was administrated at the same dose.

### 4.2. Preclinical Model of Perianal Fistulizing Crohn’s Disease

**Figure 5:**
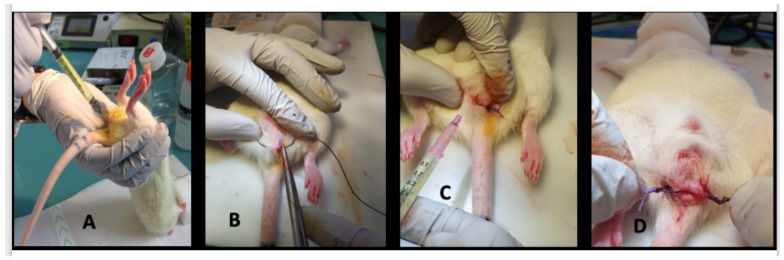
Main steps of fistula formation (day 7). (**A**): rectal enema with 500 µL of TNBS solution. (**B**): insertion of a surgical thread (Vicryl^®^ 1, ETHICON Laboratory, Issy les Moulineaux, France) into the rectum (internal orifice) and exiting at the perineum about 1 cm from the anal margin (external orifice). (**C**): instillation of 100 µL of a TNBS solution within each fistula tract. (**D**): 2 fistulas were created at 3 o’clock and 9 o’clock.

**Figure 6:**
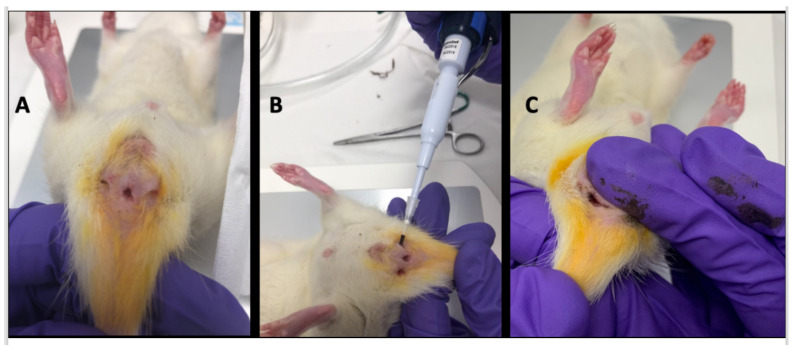
Treatment with USPIO. (**A**): inflammatory fistulas obtained at day 35. (**B**): treatment with 2 µL of USPIO injected directly into the fistula tract. (**C**): external manual pressure on the perineum maintained for 1 min in order to close the tract.

The following protocol is summarized in Figure 4:

- day 0, proctitis induction: proctitis was induced with a 500 µL rectal enema containing a TNBS solution (2,4,6-trinitrobenzenesulfonic acid solution, picrylsulfonic acid, SIGMA laboratory). The TNBS solution contained 85.3 µL of TNBS (i.e., 25 mg), 250 µL of 100% ethanol, and 164.7 µL of saline solution. The rectal enema was maintained for at least 1 min (Figure 5A). The rectal inflammatory peak was reached between day 5 and day 7 after the enema.
- day 7, fistula formation: at the peak of the rectal inflammation, 2 fistulas were created on each rat. The rats were placed in a supine position and this position was the reference to locate the fistulas (scrotum at 12 o’clock, tail at 6 o’clock). As shown in Figure 5B–D, the fistulas were created at 3 o’clock and 9 o’clock by inserting a surgical suture (Vicryl^®^ 1, Ethicon Laboratory) through the rectum (internal orifice) and exiting at the perineum about 1 cm from the anal margin (external orifice). In order to obtain a good caliber, the fistula tract was sharpened with the surgical suture. An 18 G blunt fill needle was passed through the fistula as well as a 10 µL filter tip. We obtained internal and external orifices of approximately 2 mm in diameter. At the end of the surgical procedure, the thread was retained in the fistula tract and 100 µL of a TNBS solution was instilled within each tract (Figure 5C). The TNBS solution contained, for a 100 µL instillation, 17.06 µL of TNBS (i.e., 5 mg), 50 µL of pure ethanol, and 32.94 µL of saline solution. This concentration was identical to the rectal enema used for proctitis induction.
- day 8 to day 34, monitoring threads in position: threads were maintained for 28 days. During this period, the rats were examined every 1 to 2 days to ensure that the fistulas were well-tolerated and that no threads fell out. If threads were lost, they were reinserted the same day during a short anesthesia. Twice a week, 100 µL of the same TNBS solution was instilled within each fistula tract.
- day 35, baseline MRI: after maintaining the threads for 28 days, 2 inflammatory fistulas were obtained (Figure 6A) and a perineal MRI was performed to assess the pre-treatment tracts.

### 4.3. Treatment with the Ultrasmall Particles of Iron Oxide

**Figure 7:**
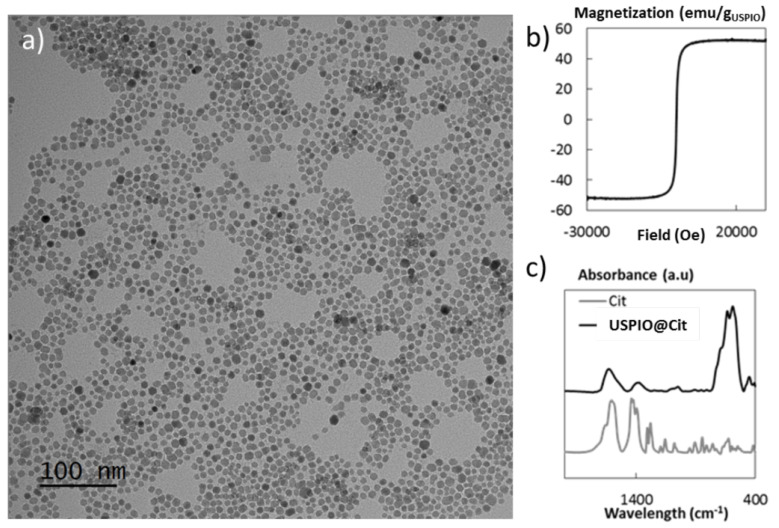
Physico-chemical characterizations of the citrated USPIO nanoparticles: (**a**) TEM image of the citrated USPIO nanoparticles of 9.0 ± 2.2 nm; (**b**) magnetization curve of the citrated USPIO nanoparticles at room temperature; and (**c**) FTIR spectra of the coated USPIO nanoparticles (USPIO@Cit) and the corresponding coating molecule (Cit).

USPIO were composed of iron oxide Fe_3_O_4_ ultrasmall superparamagnetic iron oxide nanoparticles (USPIO-NP) synthesized using a microwave non-aqueous sol-gel method [24]. The USPIO-NP surface was functionalized with citrate ligands, leading to a stable USPIO suspension at pH 7.0. The magnetic core size, measured by TEM, is equal to 9.0 ± 2.2 nm (Figure 7a), and presents a superparamagnetic behavior with a saturate magnetization of 51 ± 2 emu/g_USPIO_ (Figure 7b); this leads to a strong T2 MRI contrast agent [24]. The citrate coating was qualitatively and quantitatively assessed by Fourier Transformed Infrared (FTIR) measurements (Figure 7c) and thermogravimetric analysis, leading to 350 citrate molecules per nanoparticle.

After the baseline MRI (day 35), surgical threads were removed and the fistula tract to be treated was randomly drawn. Two µL of USPIO was injected into the fistula tract (Figure 6B). In order to close the tract, external pressure on the perineum was maintained for 1 min (Figure 6C). The control fistula tract was treated with 2 µL of a saline solution.

### 4.4. Follow-Up and Fistula Assessment Methods

**Figure 8:**
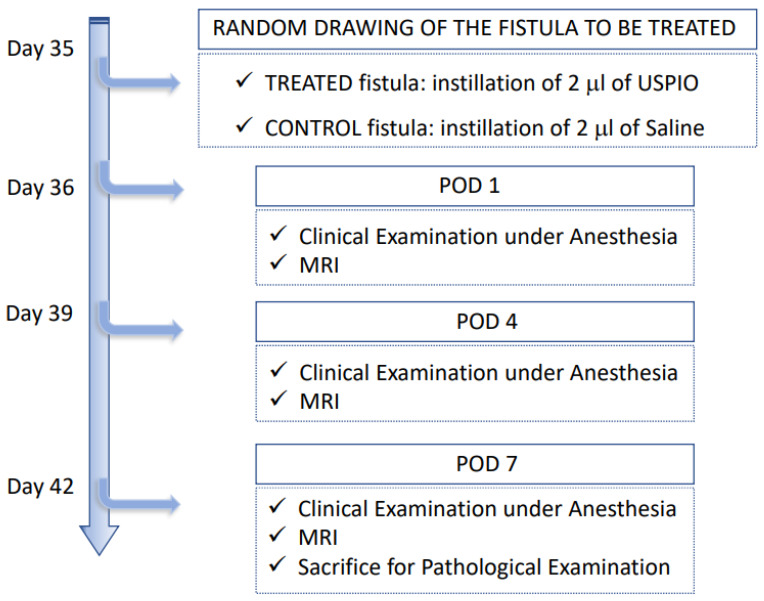
Scheme of the follow-up after treatment with saline or USPIO, corresponding to POD 0, 1, 4, and 7.

The follow-up after treatment is summarized in Figure 8. The rats were assessed at POD 1, POD 4, and POD 7. For each assessment, the rats were clinically examined under general anesthesia and underwent a perineal MRI.

The main points of clinical examination were defined according to the CDAI (Crohn’s Disease Activity Index) and PDAI (Perineal Disease Activity Index) scores: behavior, appearance, rectal bleeding, stool consistency, body weight and weight loss, and the swelling/induration/infiltration of the perineum. The clinical diagnosis of the fistula was defined by the presence of an external orifice and/or an internal orifice. The diagnosis of rectitis was defined by the presence of ano-rectal inflammation and/or bloody diarrhea.

**Figure 9:**
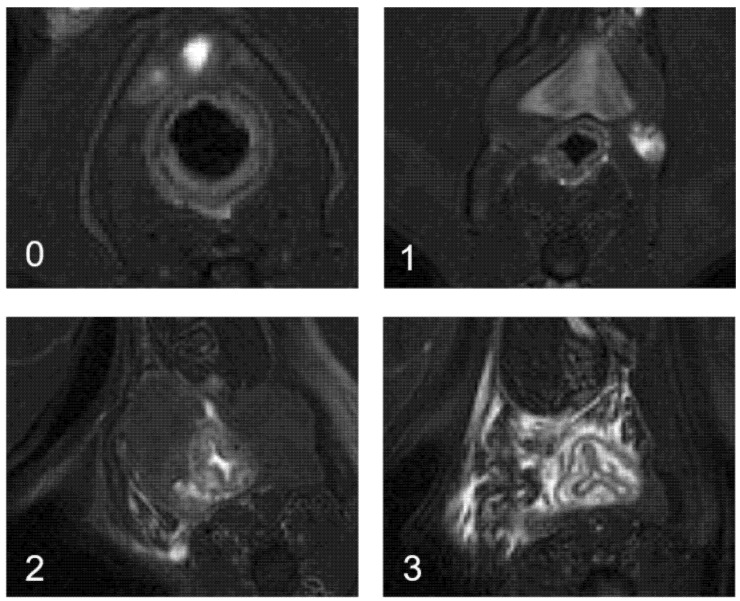
Global proctitis scores (GPS) from T2-weighted morphological images by MRI. 0: no proctitis, no inflammation in the rectal wall, nonmodified signal; 1: mild proctitis, hypersignal in the rectal wall; 2: moderate proctitis, hypersignal in the rectal wall + peri-digestive oedema; and 3: severe proctitis, hypersignal in the rectal wall + peri-digestive oedema + submucosal oedema with cocarde appearance.

Perineal MRI was performed on a 7T MRI system for small animals (Bio Spec, Bruker BioSpin, Ettlingen, Germany). The animal anesthetized with isoflurane was placed in an MRI cradle in a prone position, with the legs inserted first into the tunnel. The isocenter was placed on the presumed location of the external orifice of the fistula (at about 0.5 cm from the anus). Morphological axial T1- and T2-weighted images were acquired with fat saturation, covering 30 mm starting from the anus. Axial ultra-short echo time (UTE) images were acquired on the same region. Finally, diffusion-weighted imaging (DWI) was performed along 3 orthogonal directions, with different b values (0, 150, 400, and 800 s/mm^2^) and fat saturation to acquire 11 slices centered on the fistula track. Acquisition parameters are detailed in Table 5. Image interpretation and parameter measurements were performed by an abdominal radiologist (14 years of experience in IBD) and two research engineers (>20 years of experience in pre-clinical MRI), who were blinded to the fistula group and to the pathological results. Each rat underwent 4 MRI sessions: the first session was performed before administration of the treatment. The other sessions were performed at days 1, 4, and 7 after treatment. The T2-weighted images from the first session were used to calculate a global proctitis score (GPS) shown in Figure 9. Briefly, inflammation location and extent, seen as a hyperintense signal on T2-weighted images, were evaluated by the radiologist. The persistence of the residual fistula after surgical thread removal was assessed during each post-treatment MRI session based on its visibility (percentage of tract seen by the radiologist), its maximal diameter (mm), and the presence of an external and internal orifice compared with the same parameters evaluated at the time of the first MRI session. This assessment was performed using the T2-weighted images in priority, and completed if necessary using the UTE images (i.e., the blurry area on the T2-weighted image, a susceptibility artefact due to the iron nanoparticles present in the administered treatment). Tissue changes associated with the fistula tract were assessed by measuring the signal intensity on T2-weighted images (arbitrary units [a.u.], normalized to the muscle signal intensity) and the apparent diffusion coefficient (ADC, 10^−3^ mm^2^/s). The areas of interest were drawn directly around the tract on 3 consecutive slices, on the T2-weighted image, or on the b0 DW image. For DWI, the trace image was calculated for each b value, and then the ADC map was calculated using a pixel-by-pixel manner with a monoexponential depiction of the signal intensity.

At POD 7, after clinical examination and MRI under general anesthesia, all the rats were euthanized by cardiac explantation during sternotomy. To obtain a monobloc resection of the anorectal junction with the fistula tracts, an abdominoperineal resection was performed through a perineal approach. Dissection was performed from the bottom to the top with a circumferential section of the perineum carrying the fistula tracts. The rectum was sectioned about 3 cm from the anus at the pelvic floor muscles. The surgical specimen was divided to separate the treated and control fistulas. The tissue samples were fixed for 24 h in formaldehyde (formal solution, neutral buffered, 10%, Sigma laboratory) and stored at 4 °C. The specimens were included in paraffin according to a standardized procedure to obtain optimal orientation and longitudinal slices of the fistula tract. Several μm-thick sections were cut with a microtome along the fistula tract. For each sample, several sections were stained with hematein eosin. The diagnosis of the fistula tract was based on the presence of the following histological criteria: a tract with a lumen from an internal orifice on the rectal mucosa to an external orifice on the perineal skin, pathological signs of proctitis, and the nature of the fistula tract was defined based on the presence or the absence of fibrosis, epithelialization, and chronic inflammation.

### 4.5. Outcomes

The primary outcome was the filling or closure of the fistula tract, including the external or internal openings by USPIO evaluated by clinical examination, MRI, and pathological examination. The secondary outcomes were the evolution of fistula tract inflammation, model reassessment through the analysis of the controlled fistulas, and the safety of USPIO.

### 4.6. Statistical Analysis

Quantitative data are expressed as a mean ± standard deviation (range). Qualitative data are reported as frequency and percentage.

## Figures and Tables

**Table 1:** Clinical and radiological characteristics of the fistulas before treatment of the 17 rats that preserved 2 fistula tracts.

	Treated Fistula	Control Fistula
	(*n* = 17)	(*n* = 17)
**Clinical characteristics before treatment**		
Rectitis		
- Soft stools	17 (100) ^a^	
- Perineal tumefaction	17 (100)	
- Rectorrhagia	10 (59)	
Weight (g)	395 ± 51 [325–521] ^b^	
External orifice presence	17 (100)	17 (100)
Internal orifice presence	17 (100)	17 (100)
**MRI characteristics before treatment**		
Fistula tract ^c^	17 (100)	17 (100)
External orifice presence	17 (100)	17 (100)
Internal orifice presence	16 (94)	17 (100)
Fistula tract diameter (mm)	2.17 ± 0.6 [1.2–3.3]	1.92 ± 0.3 [1.5–2.5]
Peripheral tract inflammation		
- T2 Signal (a.u)	3.60 ± 0.56	3.50 ± 0.52
	[2.22–4.56]	[2.13–4.61]
- ADC (mm^2^/s)	1.42 ± 0.20	1.49 ± 0.23
	[1.058–1.709]	[1.09–1.92]

^a^ Number of cases (percentage of cases). ^b^ Mean ± standard deviation [range]. ^c^ Fistula tract: the number of rats with more than 50% of visibility of the fistula tract.

**Table 2:** Radiological characteristics before treatment of the 3 rats with 1 fistula tract and the MRI findings at POD 7 and POD 14.

	USPIO Rats	Control Rat
	(*n* = 2)	(*n* = 1)
**MRI Characteristics before Treatment**		
Fistula tract ^a^	2 (100) ^b^	1 (100)
External orifice presence	2 (100)	1 (100)
Internal orifice presence	2 (100)	1 (100)
Fistula tract diameter (mm)	1.3 [0.9–1.7] ^c^	1.9
Peripheral tract inflammation		
- T2 Signal (a.u)	4.96 [3.96–5.96]	3.37
- ADC (mm^2^/s)	1.10 [0.91–1.28]	1.40
**POD 7**		
External orifice closure	2 (100)	0
Internal orifice closure	2 (100)	0
Filling/closing of fistula tract ^d^	2 (100)	0
Fistula tract diameter (mm)	4.95 [4.7–5.2]	2.3
Peripheral tract inflammation		
- T2 Signal (a.u)	NM	3.17
- ADC (mm^2^/s)	NM	0.997
**POD 14**		
External orifice closure	2 (100)	0
Internal orifice closure	2 (100)	0
Filling/closing of fistula tract	2 (100)	0
Fistula tract diameter (mm)	4.15 [3.8–4.5]	4
Peripheral tract inflammation		
- T2 Signal (a.u)	NM	2.66
- ADC (mm^2^/s)	NM	1.39

^a^ Fistula tract: the number of rats with more than 50% of visibility of the fistula tract. ^b^ Number of cases (percentage of cases). ^c^ Mean [observed values]. ^d^ Filling/closing of the fistula tract: the number of fistula tracts filled or closed ≥ 50% by either USPIO or saline. NM: non-measurable; POD: post-operative day; USPIO rats: 2 rats with only 1 fistula tract treated by USPIO; Control rat: 1 rat with only 1 fistula tract treated by saline.

**Table 3:** Clinical evolution of the fistula tracts.

	Treated Fistula (*n* = 17)	Control Fistula (*n* = 17)
**External orifice closed**		
POD 1	17 (100) ^a^	3 (17)
POD 4	17 (100)	15 (88)
POD 7	17 (100)	15 (88)
**Internal orifice closed**		
POD 1	17 (100)	17 (100)
POD 4	17 (100)	17 (100)
POD 7	17 (100)	17 (100)
**Weight (g)**		
POD 1	395 ± 51 [325–521] ^b^	
POD 4	407 ± 53 [329–534]	
POD 7	414 ± 54 [337–538]	

^a^ Number of cases (percentage of cases). ^b^ Mean ± standard deviation [range].

**Table 4:** MRI findings of the fistulas after treatment of the 17 rats that preserved 2 fistula tracts.

	Treated Fistula	Control Fistula
	(*n* = 17)	(*n* = 17)
**POD 1**		
External orifice closure	17 (100) ^a^	3 (17)
Internal orifice closure	17 (100)	0
Filling/closing of fistula tract ^b^	17 (100)	0
Fistula tract diameter (mm)	3.7 ± 0.9 [2.2–5.4] ^c^	1.8 ± 0.5 [1.2–2.9]
Peripheral tract inflammation		
- T2 Signal (a.u)	NM	3.14 ± 0.5 [2.4–4.29]
- ADC (mm^2^/s)	NM	1.54 ± 0.19 [1.20–1.94]
**POD 4**		
External orifice closure	17 (100)	7 (41)
Internal orifice closure	17 (100)	2 (11)
Filling/closing of fistula tract	17 (100)	3 (17)
Fistula tract diameter (mm)	3.2 ± 0.8 [1.9–4.5]	1.6 ± 0.4 [1.1–2.4]
Peripheral tract inflammation		
- T2 Signal (a.u)	NM	2.49 ± 0.5 [1.79–3.46]
- ADC (mm^2^/s)	NM	1.55 ± 0.29 [1.21–2.36]
**POD 7**		
External orifice closure	17 (100)	9 (53)
Internal orifice closure	17 (100)	4 (23)
Filling/closing of fistula tract	17 (100)	5 (29)
Fistula tract diameter (mm)	3.2 ± 0.9 [1.2–4.8]	1.4 ± 0.3 [0.9–2]
Peripheral tract inflammation		
- T2 Signal (a.u)	NM	2.25 ± 0.5 [1.64–3.35]
- ADC (mm^2^/s)	NM	1.49 ± 0.27 [1.14–1.96]

^a^ Number of cases (percentage of cases). ^b^ Filling/closing of the fistula tract: the number of fistula tracts filled or closed ≥ 50% by USPIO or saline. ^c^ Mean ± standard deviation [range]. NM: non-measurable; POD: post-operative day.

**Table 5:** MRI acquisition parameters.

	T1-Weighted	T2-Weighted	UTE	DWI
**Echo time (ms)**	3.8	56	0.008	23
**Repetition time (ms)**	460	5300	4	2000
**Number of averages**	2	3	1	1
**Other specific parameters**	FLASH sequence	RARE sequence	3D acquisition	20 segments; 3 directions; b values = 0, 150, 400, 800 s/mm^2^
**Field of view (mm)**	60 × 60	60 × 60	60 × 60 × 60	60 × 60
**Matrix**	256 × 256	256 × 256	128 × 128 × 128	128 × 128
**Slice thickness (mm)**	1	1	-	1
**Number of slices**	29	29	-	11
**Fat saturation**	Yes	Yes	No	Yes
**Acquisition time**	3 min 55 s	8 min 29 s	3 min 25 s	6 min 40 s

## Data Availability

The data presented in this study are available on request from the corresponding author.
